# Dr. George Roberts

**Published:** 1893-02

**Authors:** 


### DR. GEORGE ROBERTS.

Dr. George Roberts died in Philadelphia, December 14, 1892, of fatty degeneration of the heart. Dr. Roberts was born in Chester County, Pa., in 1824. He began his dental work with Dr. John Anderson, of Kennett Square, Pa. He subsequently removed to Talbotton, Ga., where he remained until the breaking out of the Rebellion. He then returned to Pennsylvania, locating in Philadelphia, and practised with his brother, Dr. Spencer Roberts, for some time; but eventually located in Arch Street, where he remained until his death.

Dr. Roberts never lost his interest in his profession, manifesting this by active work in his local society,—the Odontological Society of Pennsylvania. It was exceedingly rare to find him absent from these monthly gatherings, and when present was always on the side of progress in the Society or profession at large. His activity was not so much in the arena of discussion, but was equally potential for good. His example of constant interest and work has been a strength to his colleagues, and should be an incentive to younger men to work while their day lasts for the advancement of their profession.

He married a daughter of Dr. Anderson at Kennett Square. She survives him, with three sons and one daughter.

